# Re-evaluating the suitability of using fluoroquinolones in the treatment of infections in the context of FQ consumption and correlating changes to microorganism resistance levels in EU/EEA countries between 2016 and 2021

**DOI:** 10.1007/s00210-023-02622-2

**Published:** 2023-07-26

**Authors:** Piotr Serwacki, Mateusz Gajda, Wioletta Świątek-Kwapniewska, Marta Wałaszek, Karolina Nowak, Jadwiga Wójkowska-Mach

**Affiliations:** 1 Department of Anesthesiology and Intensive Care, St. Luke’s Provincial Hospital, Lwowska 178A, 33-100 Tarnów, Poland; 2 Department of Microbiology, Faculty of Medicine, Jagiellonian University Medical College, 18 Czysta Street, 31-121 Kraków, Poland; 3 Academy of Science in Tarnów - Public University in Tarnów, Mickiewicza 8, 33-100 Tarnów, Poland; 4 5th Military Hospital with Policlinic, Wrocławska 1-3, 30-901 Kraków, Poland

**Keywords:** Antibiotic consumption, Antimicrobial resistance, European Union, Fluoroquinolones, Poland

## Abstract

The aim of this study was to re-evaluate fluoroquinolone (FQ) use in treatment by analyzing the consumption of FQ and the resistance levels of selected Gram‑negative bacilli, as well as *Neisseria gonorrhoeae* and *Mycobacterium tuberculosis complex* in EU/EEA countries and in Poland between 2016 and 2021. Data from ECDC surveillance systems EARS-Net, Euro-GASP, and the European Tuberculosis Surveillance Network were included in the description of pathogen resistance. And the ESAC-Net project for determining FQ consumption. In over half of the EU/EEA countries, the consumption of fluoroquinolones decreased in both the community sector and the hospital sector between 2016 and 2021. The prevalence of FQ-R *Escherichia coli*, *Klebsiella pneumoniae*, *Acinetobacter* spp., *Mycobacterium tuberculosis complex*, and *Neisseria gonorrhoeae* exceeded 20%. The prevalence of fluoroquinolone-resistant (FQ-R) *Pseudomonas aeruginosa* in EU/EEA countries was less than 20% except for 2017. In most EU/EEA countries, the use of FQ has reduced in last years, except for countries like Poland where FQ were an overused group of antibiotics in the treatment of various types of infections. Fluoroquinolones, as life-saving antibiotics in severe infections, should be withdrawn from empirical treatment in Poland and in countries with a high prevalence of FQ-R microorganisms.

**Supplementary information:**

The online version contains supplementary material available at 10.1007/s00210-023-02622-2.

## Introduction

Fluoroquinolones (FQs) represent a significant breakthrough in the field of infectious disease medicine since their introduction to the market in the 1970s. Their broad-spectrum activity made them an attractive option for empirical treatment of common infections when the etiological agent was uncertain. However, according to World Health Organization (WHO), the emergence of FQ-resistant microorganisms such as *Neisseria gonorrhoeae* poses a serious threat to the classification of microorganisms because of the need for new antibiotics (Geneva: World Health Organization 2019).

The mechanism of action of FQs involves restraining enzymes such as topoisomerase IV and gyrase (topoisomerase II). This results in the inhibition of DNA or RNA synthesis and a separation of their strands. Finally, the accumulation of damaged DNA in bacterial cells leads to apoptosis (Hooper and Jacoby 2016; Naeem et al. 2016). Fluoroquinolones derive from the quinolone group of antibiotics (generation I). The fluoridation of the quinolone molecule at position C6 is a crucial step in the synthesis process (Hooper and Jacoby 2016; Naeem et al. 2016; Postma et al. 2015; Markiewicz et al. 2021). The first fluorinated derivative, flumequine (generation I), was patented in 1973. Subsequent generation II members such as norfloxacin (patented in 1973 and introduced to the market in 1987) and ciprofloxacin (patented in 1981 and introduced to the market in 1986) showed improved activity against Gram-negative bacteria, a very important group of microorganisms associated with the most common infections, as well as atypical bacteria. Subsequent generations of FQs (III and IV) were modified to increase their activity against Gram-positive bacteria and anaerobic bacteria (Hooper and Jacoby 2016; Postma et al. 2015; Sharma et al. 2009). Some of the newer FQs such as levofloxacin, moxifloxacin, and gatifloxacin have excellent pharmacological properties, particularly in the treatment of lower respiratory tract infections, due to their ability to penetrate and achieve high concentrations in lung tissues (Postma et al. 2015; Karampela and Dalamaga 2020; Kaysin and Viera 2016).

As a result, a group of antibiotics was created that are willingly used in the treatment of primarily community and healthcare-associated pneumonia (PNU), community and healthcare-associated urinary tract infection (UTI), and various forms of sexually transmitted infections (STI) (Naeem et al. 2016).

Despite their broad antimicrobial spectrum, using FQs is inseparably linked with major side effects, e.g., increased risk of acute valve insufficiency, aortic dissection or aortic aneurysm, tendon rupture, peripheral neuropathy, or hypoglycemic coma. For this reason, both the American Food and Drug Administration (FDA) (Food and Administration 2016, 2018a, 2018b, 2018c) and the European Medicines Agency (EMA) (European Medicines Agency 2019) have issued several warnings regulating the use of FQs in recent years (i.e., 2016–2019), both in systemic and inhalational form. The EMA official warning states that FQs should not be used:

- To treat infections that might get better without treatment or are not severe (such as throat infections).
- To treat non-bacterial infections, e.g., non-bacterial (chronic) prostatitis.
- To prevent traveler’s diarrhea or recurring lower urinary tract infections (urine infections that do not extend beyond the bladder).
- To treat mild or moderate bacterial infections unless other antibacterial medicines commonly recommended for these infections cannot be used (European Medicines Agency 2019).

Furthermore, the improper use of antibiotics carries negative individual effects for patients who undergo such treatment. The effects result directly from the properties of the substance used—the adverse effects of the drug or the consequence of its primary action, e.g., changes in a patient's microbiota. The most common effect of intestinal dysbiosis is a *Clostridioides difficile* infection (CDI) that occurs during or after treatment with antibiotics, especially those with broad-spectrum activity, such as fluoroquinolones (Jachowicz et al. 2020; Centers for Disease Control and Prevention 2022).

Simultaneously, numerous systemic, transnational studies on antimicrobial resistance and the use of antibiotics are being conducted. Due to their scope, the most significant are the surveillance systems conducted by the European Centre for Disease Prevention and Control (ECDC). In the European Union/European Economic Area (EU/EEA), several projects function to monitor the spread and changes in drug susceptibility of pathogens: the European Antimicrobial Resistance Surveillance Network (EARS-Net) for *Escherichia coli*, *Klebsiella pneumoniae*, *Pseudomonas aeruginosa*, *Acinetobacter* spp., the European Gonococcal Antimicrobial Surveillance Program (Euro-GASP) for *Neisseria gonorrhoeae*, and the European Tuberculosis Surveillance Network for *Mycobacterium tuberculosis*. Antibiotic consumption is reported in the European Surveillance Antibiotic Consumption Network (ESAC-Net) coordinated by the ECDC. Data is updated annually.

Benchmarking of surveillance data for healthcare management, including the prevalence of antibiotic resistance and antibiotic consumption, is used to inform prevention strategies and improve patient safety. The aim of this study was to determine the place of FQ in contemporary antimicrobial therapy, particularly in empirical therapy. This was achieved based on the analysis of fluoroquinolone antibiotic consumption and the prevalence of fluoroquinolone-resistant (FQ-R) selected microorganisms, including *E. coli*, *Klebsiella pneumoniae*, *Pseudomonas aeruginosa*, *Acinetobacter* spp., *Neisseria gonorrhoeae*, and *Mycobacterium tuberculosis*, in EU/EEA countries, including Poland, from 2016 to 2021. The period after the EMA recommendations on limiting the use of FQ, i.e., 2019–2021, which included the first two years of the COVID-19 pandemic was particularly considered. Selected microorganisms are or were closely related to the use of fluoroquinolones, often as a first-line treatment for infections they cause. It is believed that the misuse of antibiotics causes selective pressure favoring the emergence and spread of resistant pathogens, and since these changes occur over the years, the analysis period of antibiotic consumption was extended to 2012–2021.

## Materials and methods

### Consumption of fluoroquinolones

Based on the ESAC-Net 2022 report on antibiotic consumption in 27 EU countries (currently, the UK is not included due to their exit from the EU) and 2 EEA countries (Iceland and Norway) (European Centre for Disease Prevention and Control 2022a) along with the attached tables (D6 and D14), the consumption of FQ in PL and EU/EEA countries was compared from 2012 to 2021. The lack of hospital-level consumption data from Cyprus and Germany is not justified in the ESAC-Net 2022 report (European Centre for Disease Prevention and Control 2022a).

Antibiotic consumption was expressed as the aggregate sum of defined daily doses (DDD) according to the ATC/DDD (Anatomical Therapeutic Chemical) system of the World Health Organization. All antibacterials designated for systemic use were included in the ECDC study. Defined daily dose (DDD) is the assumed average maintenance dose of a drug used by adults for approved indications per day. Fluoroquinolones have the ATC code J01M and are a subgroup of quinolone and quinoxaline antibacterials (World Health Organization 2023).

### Microorganism resistance

The study included microorganisms for which resistance to FQ was monitored by ECDC surveillance from 2016 to 2021:

- Selected Gram-negative bacilli: *Escherichia coli*, *Klebsiella pneumoniae*, *Pseudomonas aeruginosa*, and *Acinetobacter* spp. isolated from invasive infections (mostly bloodstream infections, but isolates from cerebrospinal fluid are reportable). Data sources: EARS-Net (European Centre for Disease Prevention and Control 2022b) and the interactive ECDC “Surveillance Atlas of Infectious Diseases” (European Centre for Disease Prevention and Control 2023). Data refers only to the resistance to ciprofloxacin and levofloxacin.
- *Neisseria gonorrhoeae* isolated from different materials and anatomical areas (genital, rectal, pharyngeal, blood, eye, joint fluid) of patients with gonorrhea. Data sources: Euro-GASP, Gonococcal antimicrobial susceptibility surveillance in the Europe Union/European Economic Area Summary of results for 2020 (European Centre for Disease Prevention and Control 2022c) and the interactive ECDC “Surveillance Atlas of Infectious Diseases” (European Centre for Disease Prevention and Control 2023). Data refers only to the resistance to ciprofloxacin.
- *Mycobacterium tuberculosis* isolated from pulmonary tuberculosis cases. Data source: ECDC and WHO “Tuberculosis surveillance and monitoring in Europe 2022” (European Centre for Disease Prevention and Control WRO for E 2022). Data covers 2016–2020 due to the lack of published data for 2021 at the time of completing this study. Data refers only to countries reporting second-line anti-TB drug susceptibility testing for at least one fluoroquinolone (ciprofloxacin, gatifloxacin, levofloxacin, moxifloxacin, and ofloxacin) and one injectable drug (amikacin, capreomycin, and kanamycin). According to the ECDC and WHO report (European Centre for Disease Prevention and Control WRO for E 2022), drug resistance surveillance methods vary across countries and local regulations. Drug susceptibility testing for second-line drugs has been conducted only on multidrug-resistant tuberculosis (MDR-TB) cases, unlike the testing for selected Gram-negative bacilli and *Neisseria gonorrhoeae* where it is performed on the total number of cases.

In order to conduct a comprehensive analysis of the prevalence of fluoroquinolone resistance among selected Gram-negative bacilli, a composite resistance index endpoint described as the “resistance rate” was introduced due to the large amount of related data. This metric was based on the average resistance observed in four Gram-negative bacilli that were available in the ECDC database. The resistance rate was calculated to provide a more comprehensive understanding of the overall resistance patterns in different locations:

$$\mathrm{Resistance}\;\mathrm{rate}=\frac{\mathrm{Resistance}\;\mathrm{for}\;\mathrm{pathogen}\;1+\;\dots\;+\mathrm{resistance}\;\mathrm{for}\;\mathrm{pathogen}\;N}N$$

Resistance rates were computed for Poland, as well as for countries that exhibited the most significant increases and decreases in FQ consumption trends. Resistance rates for countries that had the lowest overall FQ consumption (which was below 0.11) were also calculated.

The statistical analysis was performed with IBM SPSS Statistics ver. 28.0.1.0. The figure was prepared with Microsoft Excel ver. 16.72.

## Results

### Consumption of FQs in Poland and Europe

In the analyzed countries, excluding Germany and Cyprus which do not report hospital-level consumption data, the consumption of FQ in hospital sector was significantly lower than in outpatient. In 2012, it was respectively 0.26 and 1.98 DDD/1000 citizens/day. After 9 years, in 2021, it was respectively 0.15 and 1.16 DDD/1000 citizens/day.

In the community sector (Table 1), consumption in 11 (39.3%) of the countries was lower than the EU/EEA average, with the lowest consumption in Norway (0.22 DDD/1000 citizens/day). The greatest decrease in consumption, over sixfold, was noted in Belgium (from 2.77 to 0.45 DDD/1000 citizens/day), while Bulgaria had the highest increase in consumption (from 2.40 to 3.92 DDD/1000 citizens/day), approximately 160%. In Poland, during the analyzed period, consumption levels were stable from 1.19 to 1.27 DDD/1000 citizens/day with a short-term increase of approximately 23% from 2015 to 2018.

**Table 1:** Trends in the consumption of quinolone antibacterials (ATC J01M) in the community sector, EU/EEA countries, and Poland, 2012–2021, expressed as DDD per 1000 inhabitants per day (based on Table D6 (European Centre for Disease Prevention and Control 2022a))

Country	2012	2013	2014	2015	2016	2017	2018	2019	2020	2021	Trend
Austria	1.30	1.47	1.30	1.31	1.20	1.23	1.04	0.73	0.57	0.52	↓
Belgium	2.77	2.64	2.55	2.57	2.40	2.17	1.16	0.57	0.46	0.45	↓
Bulgaria	2.40	2.52	2.87	2.83	2.78	2.86	2.83	2.76	3.35	3.92	↑
Croatia	1.49	1.47	1.50	1.50	1.49	1.50	1.48	1.36	1.22	1.44	NC
Czechia	1.03	0.85	0.88	0.88	n/d	n/d	n/d	n/d	n/d	0.48	N/A
Denmark	0.55	0.52	0.50	0.49	0.48	0.44	0.41	0.37	0.33	0.32	↓
Estonia	0.85	0.89	0.90	0.92	0.85	0.79	0.75	0.65	0.72	0.59	↓
Finland	0.89	0.84	0.82	0.73	0.74	0.67	0.62	0.47	0.39	0.36	↓
France	1.92	1.84	1.75	1.60	1.51	1.37	1.30	1.21	1.09	0.99	↓
Germany	1.45	1.42	1.34	1.33	1.24	1.11	0.96	0.63	0.49	0.43	↓
Greece	2.36	2.06	2.56	2.64	2.61	2.60	2.94	3.04	2.61	2.16	NC
Hungary	1.97	2.11	2.43	2.71	2.38	2.40	2.31	1.93	1.41	1.42	NC
Iceland	n/d	n/d	0.88	0.92	0.92	0.82	0.83	0.57	0.49	0.45	↓
Ireland	0.87	0.87	0.84	0.92	0.87	0.81	0.76	0.55	0.42	0.38	↓
Italy	3.48	3.55	3.41	3.37	3.23	2.68	2.65	1.99	1.67	1.62	↓
Latvia	1.03	1.05	1.05	1.05	1.05	1.03	0.94	0.85	0.77	0.71	↓
Lithuania	1.00	0.96	0.91	0.91	0.88	0.87	0.88	0.82	0.73	0.66	↓
Luxembourg	2.77	2.65	2.57	2.48	2.41	2.77	2.05	1.57	1.27	1.26	N/A
Malta	2.01	2.92	3.06	2.64	2.37	2.18	2.27	1.89	1.36	1.31	↓
Netherlands	0.81	0.76	0.79	0.77	0.75	0.73	0.73	0.67	0.64	0.64	↓
Norway	0.56	0.54	0.50	0.46	0.41	0.35	0.32	0.28	0.24	0.22	↓
^**a**^**Poland**	**1.19**	**1.18**	**1.21**	**1.40**	**1.42**	**1.49**	**1.48**	**1.35**	**1.15**	**1.27**	**NC**
Portugal	2.47	2.18	2.12	2.05	1.92	1.75	1.71	1.54	1.21	1.17	↓
Romania	n/d	n/d	n/d	n/d	n/d	n/d	n/d	3.12	2.96	3.17	N/A
Slovakia	1.95	2.18	0.63	2.40	2.26	2.12	2.07	1.47	1.14	1.17	NC
Slovenia	1.08	1.10	1.11	1.16	1.14	1.11	1.11	0.99	0.84	0.85	↓
Spain	2.46	2.36	2.31	2.35	2.90	2.82	2.71	2.30	1.78	1.72	N/A
Sweden	0.75	0.71	0.69	0.68	0.66	0.63	0.61	0.56	0.49	0.49	↓
^**a, b**^**EU/EEA**	**1.98**	**1.96**	**1.92**	**1.93**	**1.88**	**1.76**	**1.68**	**1.40**	**1.20**	**1.16**	**↓**

Abbreviations: *NC*, no change; *N/A*, not applicable; *n/d*, no data. Trend analysis was not performed because of missing data, changes in the type of data, or changes in data process—see reference (European Centre for Disease Prevention and Control 2022a)

^a^Rows with Poland and EU/EEA results were bolded

^b^EU/EEA refers to the population-weighted mean consumption based on reported or imputed antimicrobial consumption data from all 29 EU/EEA countries and excludes the UK. Country adjustments were applied—see reference(European Centre for Disease Prevention and Control 2022a)

In the hospital sector (Table 2), 14 (51.8%) countries showed lower FQ consumption than the EU/EEA average, the lowest (0.03 DDD/1000 citizens/day), again, in Norway. The greatest consumption decrease, over threefold, was noted in Latvia (from 0.30 to 0.11 DDD/1000 citizens/day). In Bulgaria and Croatia, consumption increases were noted—the greatest in Bulgaria for approx. 283% starting value (from 0.12 to 0.34 DDD/1000 citizens/day). In Poland, during the analyzed period, consumption levels were stable from 0.15 to 0.16 DDD/1000 citizens/day.

**Table 2:** Trends in consumption of quinolone antibacterials (ATC J01M) in the hospital sector, EU/EEA countries, and Poland, 2012–2021, expressed as DDD per 1000 inhabitants per day (based on Table D14 (European Centre for Disease Prevention and Control 2022a))

Country	2012	2013	2014	2015	2016	2017	2018	2019	2020	2021	Trend
Austria	n/d	n/d	n/d	n/d	n/d	n/d	n/d	0.17	0.13	0.12	N/A
Belgium	0.23	0.22	0.21	0.21	0.20	0.19	0.17	0.16	0.13	0.13	↓
Bulgaria	0.12	0.11	0.14	0.14	0.15	0.14	0.17	0.17	0.33	0.34	↑
Croatia	0.19	0.19	0.20	0.21	0.21	0.23	0.24	0.24	0.20	0.24	↑
Czechia	n/d	n/d	n/d	n/d	n/d	n/d	n/d	n/d	n/d	0.12	N/A
Denmark	0.19	0.19	0.18	0.17	0.15	0.14	0.13	0.12	0.11	0.10	↓
Estonia	0.30	0.19	0.18	0.15	0.14	0.20	0.11	0.13	0.16	0.11	↓
Finland	0.30	0.29	0.29	0.26	0.25	0.22	0.20	0.15	0.16	0.12	↓
France	0.26	0.25	0.24	0.23	0.22	0.19	0.23	0.17	0.16	0.17	↓
Greece	0.21	0.18	0.22	0.26	0.21	0.20	0.22	0.20	0.19	0.20	NC
Hungary	0.22	0.21	0.21	0.21	0.21	0.21	0.19	0.14	0.14	0.12	↓
Iceland	n/d	n/d	n/d	n/d	n/d	0.09	0.08	0.05	0.04	0.05	N/A
Ireland	0.11	0.11	0.10	0.11	0.10	0.10	0.10	0.08	0.06	0.05	↓
Italy	0.43	0.42	0.40	0.43	0.40	0.39	0.36	0.26	0.21	0.15	↓
Latvia	0.30	0.31	0.30	0.27	0.26	0.24	0.26	0.23	0.15	0.11	↓
Lithuania	0.37	0.17	0.17	0.28	0.27	0.24	0.18	0.19	0.19	0.12	NC
Luxembourg	0.24	0.23	0.21	0.19	0.21	0.19	0.16	0.14	0.12	0.12	N/A
Malta	0.21	0.24	0.37	0.47	0.30	0.30	0.33	0.23	0.28	0.16	NC
Netherlands	0.11	0.10	0.10	0.10	0.09	0.09	0.08	0.07	0.07	0.06	↓
Norway	0.08	0.07	0.07	0.06	0.05	0.05	0.04	0.04	0.03	0.03	↓
^**a**^**Poland**	n/d	n/d	**0.15**	**0.15**	**0.20**	**0.17**	**0.18**	**0.16**	**0.15**	**0.16**	**NC**
Portugal	0.09	0.16	0.15	0.14	0.13	0.11	0.11	0.10	0.09	0.09	NC
Romania	n/d	n/d	n/d	n/d	n/d	n/d	n/d	0.20	0.17	0.16	N/A
Slovakia	0.25	0.27	0.30	0.31	0.29	0.28	0.27	0.20	0.18	0.19	↓
Slovenia	0.21	0.20	0.21	0.22	0.21	0.20	0.19	0.17	0.13	0.13	↓
Spain	n/d	n/d	n/d	n/d	0.34	0.30	0.27	0.23	0.19	0.17	N/A
Sweden	0.16	0.16	0.16	0.16	0.16	0.15	0.14	0.13	0.13	0.13	↓
^**a, b**^**EU/EEA**	**0.26**	**0.26**	**0.25**	**0.25**	**0.24**	**0.22**	**0.23**	**0.18**	**0.16**	**0.15**	**↓**

Abbreviations: *NC*, no change; *N/A*, not applicable; *n/d*, no data. Trend analysis was not performed because of missing data, changes in the type of data, or changes in data process—see reference (European Centre for Disease Prevention and Control 2022a)

^a^Rows with Poland and EU/EEA results were bolded

^b^EU/EEA refers to the population-weighted mean consumption based on reported or imputed antimicrobial consumption data from the 21 EU/EEA countries that reported hospital sector data for the entire 10 years and excludes the UK

### Resistance levels

#### Gram-negative bacilli

Nearly all analyzed selected Gram-negative bacilli have exceeded the FQ 30% population-weighted mean resistance threshold over the entire period considered, both in EU/EEA countries and in Poland, with the exception of *Escherichia coli* and *Pseudomonas aeruginosa* in EU/EEA countries (see Appendix I).

**Fig. 1:**
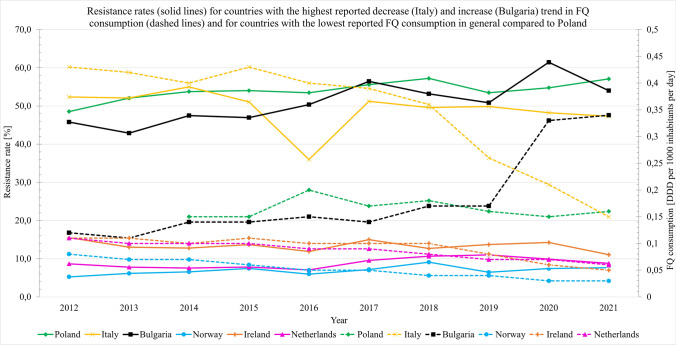
Resistance rates (solid lines) for countries with the highest reported decrease (Italy) and increase (Bulgaria) trend in FQ consumption, and for countries with the lowest reported FQ consumption—below 0.11 (Norway, the Netherlands, and Ireland) compared to Poland. Adapted from (European Centre for Disease Prevention and Control 2022a, 2023) $\mathrm{Resistance rate}= \frac{\mathrm{Resistance for pathogen }1+\dots +\mathrm{resistance for pathogen }N}{N}$

To illustrate the range/variability of fluoroquinolone resistance across different regions, resistance rates were calculated in the countries with the greatest reduction (Italy) and greatest increase (Bulgaria) in fluoroquinolone consumption trends. The results are compared with the data for Poland and countries with the lowest FQ consumption rates (*p* < 0.12, Fig. 1).

The prevalence of FQ-R microorganisms in Poland has increased more than twofold in the case of *Klebsiella pneumoniae* (70.4% in 2021), *Acinetobacter* spp. (92.6% in 2021), and *Pseudomonas aeruginosa* (32.3% in 2021) when compared to EU/EEA countries, respectively: 33.6%, 43.0%, and 18.7%. In 2021, a slightly lower difference in the prevalence of FQ-R was observed in *Escherichia coli*. In Poland and in EU/EEA countries, respectively: 33.1% and 21.9% (see Appendix I).

#### Neisseria gonorrhoeae

In EU/EEA countries, the prevalence of FQ-R *Neisseria gonorrhoeae* (only ciprofloxacin susceptibility testing) was 63.2% in 2021 which was higher than in 2016 (46.8%). Whereas, in Poland, the prevalence of FQ-R has decreased from 57.1% in 2016 to 47.8% in 2020 and then increased to 50% in 2021 (Table 3).

**Table 3:** Total ciprofloxacin tested Neisseria gonorrhoeae isolates (N) of confirmed cases and prevalence of ^a^fluoroquinolone resistant isolates (%) in the EU/EEA and Poland, 2016–2021 (based on (European Centre for Disease Prevention and Control 2023; European Centre for Disease Prevention and Control 2022c))

Region	ABX	2016	2017	2018	2019	2020	2021
		*N* (%)	*N* (%)	*N* (%)	*N* (%)	*N* (%)	*N* (%)
^b^EU/EEA	CFx	2811 (46.8)	3247 (46.5)	3299 (50.3)	4164 (57.3)	3291 (57.7)	3598 (63.2)
Polska	CFx	77 (57.1)	65 (76.9)	73 (50.7)	53 (64.2)	23 (47.8)	14 (50)

Abbreviations: *ABX*, antimicrobial; *CFx*, ciprofloxacin

^a^Defined as fluoroquinolone-resistant if tested and interpreted as resistant (R) to ciprofloxacin in accordance with the clinical breakpoints, defined by the European Committee on Antimicrobial Susceptibility Testing (EUCAST) and the EU case definition for AMR

^b^EU/EEA refers to data reported from 27 EU/EEA countries. Germany does not report gonorrhea surveillance data. Austria has not reported data since 2014

#### Mycobacterium tuberculosis complex

In EU/EEA countries, the prevalence of FQ-R *Mycobacterium tuberculosis complex* was 27.6% in 2020 so it was a lower value than in 2016 (34.6%). Whereas, in Poland, the prevalence of FQ-R increased more than twofold from 34.2% in 2016 to 85.7% in 2020 (Table 4).

**Table 4:** Total number of pulmonary RR/MDR-TB (N) isolates and prevalence of fluoroquinolone-resistant isolates (%)—^a^pre-XDR-TB—in the EU/EEA and Poland, 2016–2020 (based on (European Centre for Disease Prevention and Control WRO for E 2022))

Region	ABX	2016	2017	2018	2019	2020
		N (%)	N (%)	N (%)	N (%)	N (%)
^b^EU/EEA	FQ^a^	879 (34.6)	904 (31.9)	812 (31.8)	602 (32.9)	417 (27.6)
Polska	FQ^a^	38 (34.2)	36 (30.6)	41 (43.9)	31 (22.6)	14 (85.7)

Abbreviations: *ABX*, antimicrobial; *FQ*, one of ciprofloxacin, gatifloxacin, levofloxacin, moxifloxacin, or ofloxacin

^a^Pre-XDR-TB—defined as TB that fulfills the definition of RR/MDR-TB (resistance to at least rifampicin) and is also resistant to any fluoroquinolone

^b^EU/EEA refers to data reported from 30 EU/EEA countries. Iceland, Lichtenstein, Luxembourg, and Malta reported no RR/MDR-TB isolates between 2016 and 2020

## Discussion

In most of the surveyed countries, the implementation of EMA and FDA recommendations regarding the restriction of fluoroquinolone use has coincided with a reduction in FQ consumption. The recommendation was mainly related to the adverse effects of FQ, but this decision is also consistent with observations regarding the growing problem of FQ-R microorganisms. The limitation in the use of FQ has been visible since 2017, where a decrease in FQ consumption in outpatient care was noted in 6 out of 28 countries. It is worth noting that the decrease in hospital settings was only noted in 2019, with a 2-year delay in relation to the introduced warnings. Despite the reduced consumption in most EU/EEA countries, the resistance of the studied microorganisms remains at a consistently high level or is increasing. As depicted in Fig. 1, Italy serves as an example of a country that has reported the greatest reduction in consumption, resulting in a decrease in resistance rates. Conversely, Bulgaria, which has reported the greatest increase in consumption during the reported period, shows an increase in resistance rates.

Countries such as Croatia, Greece, Hungary, Slovakia, and Poland (Fig. 1), despite increasing pathogen resistance and EMA and FDA warnings, not only did not ultimately reduce fluoroquinolone consumption in outpatient care, but also recorded several years of increased consumption between 2012 and 2021. Similar changes were noted in closed healthcare settings in Greece, Malta, and Slovakia.

Clinically, antibiotic treatment is most often empirical because most non-critical infections are diagnosed and treated in outpatient settings, where full microbiological diagnostics, including pathogen identification and drug susceptibility testing, are not always performed. Therefore, the literature on FQ resistance in the most common outpatient infections, such as PNU, UTI, and STI, is very scarce. In the Polish study by Stefaniuk et al. in 2013 (Stefaniuk et al. 2016), the resistance of *Enterobacteriaceae* bacilli, including *E. coli* isolated from community-acquired UTI cases, affected around 40% of strains, while according to Jurałowicz et al., in cases of outpatient recurrent UTI, 40% of *E. coli* isolates and 55% of *K. pneumoniae* isolates were ciprofloxacin-resistant (Jurałowicz et al. 2020). In another study by Ny et al. (data from 2015 to 2017 from Russia) ¼ of *E. coli* isolates from patients with UTI were ciprofloxacin-resistant (Ny et al. 2019). According to Mlynarczyk-Bonikowska et al. in Poland (2010–2012), 61% of *N. gonorrhoeae* isolates were resistant to ciprofloxacin. Nowadays, the epidemiological situation of FQ-R *N. gonorrhoeae* in Poland generally has not changed (Mlynarczyk-Bonikowska et al. 2014).

In cases of low consumption, expressed as less than or equal to 0.11 DDD per 1000 inhabitants per day, resistance may, as a result, be reduced below 18% as shown in Fig. 1. However, from the present analysis, it is difficult to observe a level of consumption that could be associated with lower resistance. The data for the Netherlands indicates that a reduction in consumption, which had been declining until 2021, did not result in significant decrease in the resistance rate.

Only in hospital conditions, with a wider availability of microbiological diagnostics, the utilization of targeted antibiotic therapy is increasing. However, even in a closed healthcare setting, it is not satisfactory. According to many authors, e.g., the Infectious Diseases Society of America and the European Society for Microbiology and Infectious Diseases guidelines, only a microbial resistance level of  < 10% is considered permissible for the empirical use of a given antibiotic in the treatment of infections mainly associated with a specific microorganism (Shah et al. 2017; Gupta et al. 2011). As shown by the above data, in the surveyed countries, all analyzed microorganisms exceeded the resistance threshold for the empirical use of fluoroquinolones (WHO Regional Office for Europe/European Centre for Disease Prevention and Control 2022; European Centre for Disease Prevention and Control 2022b, 2023; European Centre for Disease Prevention and Control 2022c; European Centre for Disease Prevention and Control WRO for E 2022).

According to a study conducted by Rostkowska et al. in Poland between October 2019 and March 2020, among 504 doctors aged 25–59 years, when evaluating the application of knowledge about antibiotic resistance of pathogens to prescribed antibiotics, it was shown that in 90% of infection treatment cases, doctors most often relied on clinical practice guidelines and their own experience. Seventy-eight percent of the 504 doctors prescribed antimicrobials at least once a week (Rostkowska et al. 2022). The two main sources of recommendations in antimicrobial therapy in Poland are the National Antibiotic Protection Program (Narodowy Program Ochrony Antybiotyków) and Practical Medicine (Medycyna Praktyczna), both freely accessible in everyday doctor practice. In the recommendations or guidelines published by them, fluoroquinolones (FQ) are recommended as first-line antimicrobials in community and healthcare-associated pneumonia, infectious exacerbations of COPD, and urinary tract infections (UTI) (Waleria and Agnieszka 2020; Filip 2017; Jan and Drabczyk 2022).

Therefore, for the invasive infections associated with the Gram-negative rods mentioned in this study, fluoroquinolones should not be used empirically in Poland and in EU/EEA countries. This applies especially to *Klebsiella pneumoniae* (resistance respectively: > 60% and  > 30% in recent years) and *Acinetobacter* spp. (resistance respectively: > 80% and  > 35% in recent years), which are microorganisms causing both hospital and community-acquired infections, notably in intensive care units. Particularly in Poland, as indicated by the presented data (Stefaniuk et al. 2016; Jurałowicz et al. 2020; Ny et al. 2019; Mlynarczyk-Bonikowska et al. 2014), there is no place for FQ in empiric treatment, especially in UTI, STI, and PNU.

The above-presented data suggest that the increasing levels of resistance of Gram-negative rods, *Neisseria gonorrhoeae*, and *Mycobacterium tuberculosis complex*, as well as the warnings and recommendations of EMA regarding the rational use of FQ, have been reflected in treatment guidelines for specific diseases and in clinical practice. An example is the WHO recommendation from 2016 and the CDC recommendation from 2021 regarding the treatment of *Neisseria gonorrhoeae* infections, where FQ use is not recommended, leaving cephalosporins as the only class of antibiotics of choice for treatment, even though FQ were previously—in 2007—indicated as first-line treatment (Walensky et al. 2021; World Health Organization 2016; St Cyr et al. 2020).

A slightly different situation is observed in the epidemiology and treatment of tuberculosis (TB), in which, according to WHO recommendations (Geneva: World Health Organization 2020) from 2020, FQs are recommended for use as additional drugs. This is due to the acquisition of resistance by strains of *Mycobacterium tuberculosis*. This concerns strains described as Hr-TB which are susceptible to rifampicin and resistant to isoniazid (most common among drug-resistant strains), RR-TB—rifampicin-resistant, MDR-TB—multi-drug resistant (resistant to at least rifampicin and isoniazid), XDR-TB—extensively drug-resistant (resistant to any FQ and  ≥ 1 of the 3 s-line drugs administered intravenously, i.e., capreomycin, kanamycin, amikacin, as well as rifampicin and isoniazid). It is precisely in relation to cases of Hr-TB in treatment that a 6-month regimen with levofloxacin (Seung et al. 2015) is recommended, while for cases of RR/MDR-TB, levofloxacin or moxifloxacin are recommended in extended regimens (Geneva: World Health Organization 2020; Seung et al. 2015). The differences in reporting second-line drug-resistant TB cases between countries, influenced by local regulations, make it difficult to apply a unified methodology with selected Enterobacterales and *N. gonorrhoeae* mentioned in this study. To ensure a uniform assessment, the standardization of surveillance and drug susceptibility testing rules is necessary.

In addition to implementing the EMA and FDA recommendations regarding FQ treatment, a study was conducted on behalf of the EMA to assess the effectiveness of the issued regulations based on an analysis of FQ consumption, prescribing patterns of FQs, and physician compliance with issued warnings regarding FQ adverse effects in the years 2016–2021 in 6 EU/EEA countries. According to the authors, the regulations had only a moderate impact on the use of FQs in clinical practice, and the observed decrease in FQ consumption began even before their issuance (Pacurariu et al. 2019).

Undoubtedly, antibiotic stewardship programs (ASPs) (CDC 2019) play a significant role in developing and adhering to the proper principles of antibiotic use, including FQ. In the USA, where the ASP idea originated, the effects of both ASPs and FDA warnings are visible in many care settings. The results constitute a lower FQ prescription rate and a re-evaluation of fluoroquinolones to be the last-line treatment in serious infections (Lin et al. 2020; Gouin et al. 2020). The importance of education for doctors in proper antibiotic use, including local training, also remains highly significant (Lin et al. 2020). Thanks to the implementation and practical application of ASP recommendations (Berrevoets et al. 2017; Mölstad et al. 2017; Patel et al. 2023; Binda et al. 2020) among EU/EEA countries, countries such as Austria, France, the Netherlands, Norway, and Sweden can boast declining trends in FQ consumption in both ambulatory and closed healthcare settings, which unfortunately does not apply to Poland.

Considering the causation between FQ usage and CDI prevalence, the authors indicate that this manuscript does not cover CDI incidence rates due to the limited availability of EU/EEA surveillance data (the latest available ECDC CDI Epidemiological Report only covers 2016 and 2017). In a study by Jachowicz et al. (Jachowicz et al. 2020) conducted from 2012 to 2018 in a hospital in southern Poland, a total of 198 CDI cases were identified, predominantly in the urology and general surgery departments. It was also found that fluoroquinolones were the most frequently used antibiotics in the urology department, and a positive correlation was confirmed between the use of fluoroquinolones and the incidence of CDI. An antibiotic stewardship intervention policy for reduced use of FQ in the hospital setting has been linked to a decrease in CDI prevalence (Lawes et al. 2017; Dingle et al. 2017). Also, the results presented by Ziolkowski et al. indicate a significant relationship between the consumption of antibiotics, including FQ, and the incidence of CDI in the ICU (Ziółkowski et al. 2018). However, it is important to note that decreased FQ consumption is not the only factor impacting CDI prevalence (Fortin et al. 2021).

One of the factors that increases the risk of high resistance of microorganisms is their improper use, for example, in surgical or procedural prophylaxis (Menz et al. 2021). Therefore, the European Commission has officially deleted the indication statement that FQ should be used as an agent of infection prevention in surgical procedures; concurrently, the European Association of Urology (EAU) was obliged to change their recommendations (Bonkat et al. 2019). Pilatz et al. suggest that fosfomycin is a good alternative to fluoroquinolones in prostate biopsy. This was confirmed in a meta-analysis that included three RCTs and two retrospective cohorts (RR = 0.2, 95% CI = 0.58–5.23, *p* = 0.00001) (Pilatz et al. 2019). In Maciejczak A. et al.’s research (Maciejczak et al. 2019), conducted between 2003 and 2014 in a single hospital in southern Poland, ciprofloxacin was used in perioperative antibiotic prophylaxis in spine surgery for patients who were allergic to beta-lactams despite the ASHP antibiotic prophylaxis guidelines from 2013 which have been recommending either clindamycin or vancomycin in neurosurgery for patients with beta-lactam allergies (Bratzler et al. 2013).

The years 2020 and 2021 coincide with the period of the SARS-CoV-2 (severe acute respiratory syndrome coronavirus 2) pandemic, which causes COVID-19. Reporting of cases of pathogens resistant to various antibiotics coincided with global changes in healthcare due to the occurrence and persistence of the COVID-19 pandemic. The pandemic had an undisputed impact on the prevention and control of infections caused by antibiotic-resistant pathogens, but unfortunately also on their spread, especially the highly resistant *Acinetobacter* spp. (Kinross et al. 2022). Despite the clinical picture of viral pneumonia, often coinciding with ARDS, during COVID-19, in many EU/EEA countries, there was no increase in the consumption of fluoroquinolones (European Centre for Disease Prevention and Control 2022a). The increasing resistance of Polish isolates may be related to the increased consumption of fluoroquinolones in Poland in 2021 due to them being frequently prescribed as an empirical broad-spectrum treatment for pneumonia in patients infected with SARS-CoV-2, but evidence is hard to come by.

In modern medicine, the loss of a group of antibiotics with such a broad spectrum of antimicrobial activity and unique pharmacological properties as fluoroquinolones due to the increasing microbial resistance is inconceivable. According to numerous authors, fluoroquinolones should be withdrawn from the empirical treatment of infectious diseases caused by microorganisms that have little or no susceptibility to fluoroquinolones. Instead, they should be restricted for targeted treatment of infections caused by microorganisms that have been shown to be susceptible to fluoroquinolones in the antibiotic susceptibility test since they constitute an invaluable life-saving group of antibiotics.

### Supplementary Information

Below is the link to the electronic supplementary material.

Supplementary file1 (XLSX 22 KB)

## Data Availability

The data that support the findings of this study are available from references.
